# Effect of blood pressure lowering medications on leg ischemia in peripheral artery disease patients: A meta-analysis of randomised controlled trials

**DOI:** 10.1371/journal.pone.0178713

**Published:** 2017-06-02

**Authors:** Diana Thomas Manapurathe, Smriti Murali Krishna, Brittany Dewdney, Joseph Vaughan Moxon, Erik Biros, Jonathan Golledge

**Affiliations:** 1 The Vascular Biology Unit, Queensland Research Centre for Peripheral Arterial Diseases, College of Medicine & Dentistry, James Cook University, Townsville, Australia; 2 Department of Vascular and Endovascular Surgery, The Townsville Hospital, Townsville, Australia; The University of Tokyo, JAPAN

## Abstract

**Background:**

It has been suggested that anti-hypertensive medications may worsen leg ischemia in peripheral artery disease (PAD) patients. We undertook a meta-analysis to assess the effect of anti-hypertensive medications on measures of leg ischemia including maximum walking distance (MWD), pain free walking distance (PFWD) and ankle brachial pressure index (ABPI). A meta-regression was performed to evaluate whether the effect of the anti-hypertensive medications on mean arterial pressure (MAP) was associated with changes in ABPI, MWD or PFWD.

**Method:**

A systematic literature search was performed to identify placebo controlled randomized control trials (RCT) testing anti-hypertensive medications, which reported baseline and follow-up measurements of: MAP and MWD, PFWD or ABPI in patients with intermittent claudication (IC) due to PAD.

**Result:**

A meta-analysis was performed on 5 RCTs comprising a total of 180 and 127 patients receiving anti-hypertensive medications and placebo respectively. This analysis suggested that anti-hypertensive medication did not significantly affect MWD, PFWD or ABPI. In contrast, the meta-regression analysis showed that the reduction in MAP due to the anti-hypertensive drugs was positively correlated with increased MWD during follow-up (β = 8.371, p = 0.035). Heterogeneity across studies, as assessed by I^2^, was high. The follow-up period within the included trials was generally short with 3 out of 5 studies having a follow-up period of ≤ 6 weeks.

**Conclusion:**

This study suggests that anti-hypertensive treatment does not worsen but may improve leg ischemia in PAD patients. Larger multicenter trials with longer anti-hypertensive treatment periods are required to clarify the effect of anti-hypertensives on leg ischemia in PAD patients.

## Introduction

Lower limb peripheral artery disease (PAD) results from narrowing and occlusion of the arteries supplying blood to the legs, usually due to atherosclerosis and associated thrombosis[1, 2]. Approximately 200 million people worldwide were estimated to have PAD in 2010, a rise of 25% since 2000[3]. The most recognised presenting complaint for PAD patients is intermittent claudication (IC). Patients with IC have significantly impaired walking ability, high rates of cardiovascular events, such as myocardial infarction and stroke, and reduced disease-related quality of life[4]. Approximately 20% of patients with PAD will die from a cardiovascular event within 5 years[5] and therefore aggressive management of cardiovascular risk factors is the primary focus of treatment[6].

Hypertension was identified by the global burden of disease study as the leading risk factor for mortality and disability-adjusted life years lost in 2010[7]. Hypertension is considered an important risk factor for PAD and its complications and approximately 50% of patients presenting with PAD are reported to have hypertension[8–12]. Clinical trials including PAD patients (in addition to those with other atherosclerosis-related diseases) have reported a relative reduction in cardiovascular events in those receiving blood pressure lowering medications of 20–30%[13, 14]. Therefore current recommendations advise that PAD patients should receive anti-hypertensive medications if their blood pressure (BP) is >140/90[15–17]. However, the use of some anti-hypertensives, particularly β blockers, has been traditionally contraindicated in PAD patients due to concerns regarding reducing peripheral perfusion[18, 19]. This opinion has been challenged by others who have reported that BP lowering medications do not worsen leg ischemia[20–22]. Some previously published randomised trials in PAD patients have reported improvements in maximum walking distance (MWD), pain free walking distance (PFWD), ankle brachial pressure index (ABPI) and calf blood flow (CBF) after commencing anti-hypertensive medications[23–30]. Other trials have reported no effect of anti-hypertensive medications on PAD patients[31, 32]. A previous Cochrane review concluded that there was lack of evidence on the efficacy of anti-hypertensive medication in patients with PAD, but did not perform a meta-analysis or meta-regression to definitely assess this[33]. In view of the current controversy regarding the effect of BP lowering medication on symptoms of PAD, we undertook a systematic review and meta-analysis to assess if anti-hypertensive medications worsen leg ischemia in PAD patients.

## Materials and methods

This meta-analysis was performed in accordance with the Preferred Reporting Items for Systematic reviews and Meta-Analyses (PRISMA) guidelines[34]. A protocol was developed following the guidelines of the PRISMA-P statement[34] and was published in the PROSPERO database (CRD42016038338).

### Search strategy

A systematic search was performed to identify placebo controlled randomised trials (RCTs) evaluating the effects of anti-hypertensive medications on three recognised measures of the severity of leg ischemia including ABPI, MWD and PFWD in patients with IC. The search strategy undertaken and the study selection criteria are detailed in S1 File.

### Data extraction

Two authors (DT and BD) extracted data independently using a predefined data extraction table (see S1 File and S1 Table for further details).

### Study quality assessment

A quality assessment tool was formulated using components from the following validated tools: a) The 25 items CONSORT 2010 checklist of information to be included when reporting a randomised trial; and b) The Cochrane collaboration tool for assessing risk of bias[35, 36]. The quality assessment details are given in S1 File.

### Quantitative data synthesis

The first author (DT) performed statistical analysis of data using the Meta-Analyst software (version beta 3.13)[37]. A description of the data synthesis is provided in S1 File.

### Assessment of publication bias

The publication bias was assessed using funnel plots and Eggers’s test using the CMA software package (Comprehensive Meta-Analysis version 3.3.070). The first author (DT) carried out the publication bias assessment.

## Results

### Characteristics of the included studies

**Fig 1:**
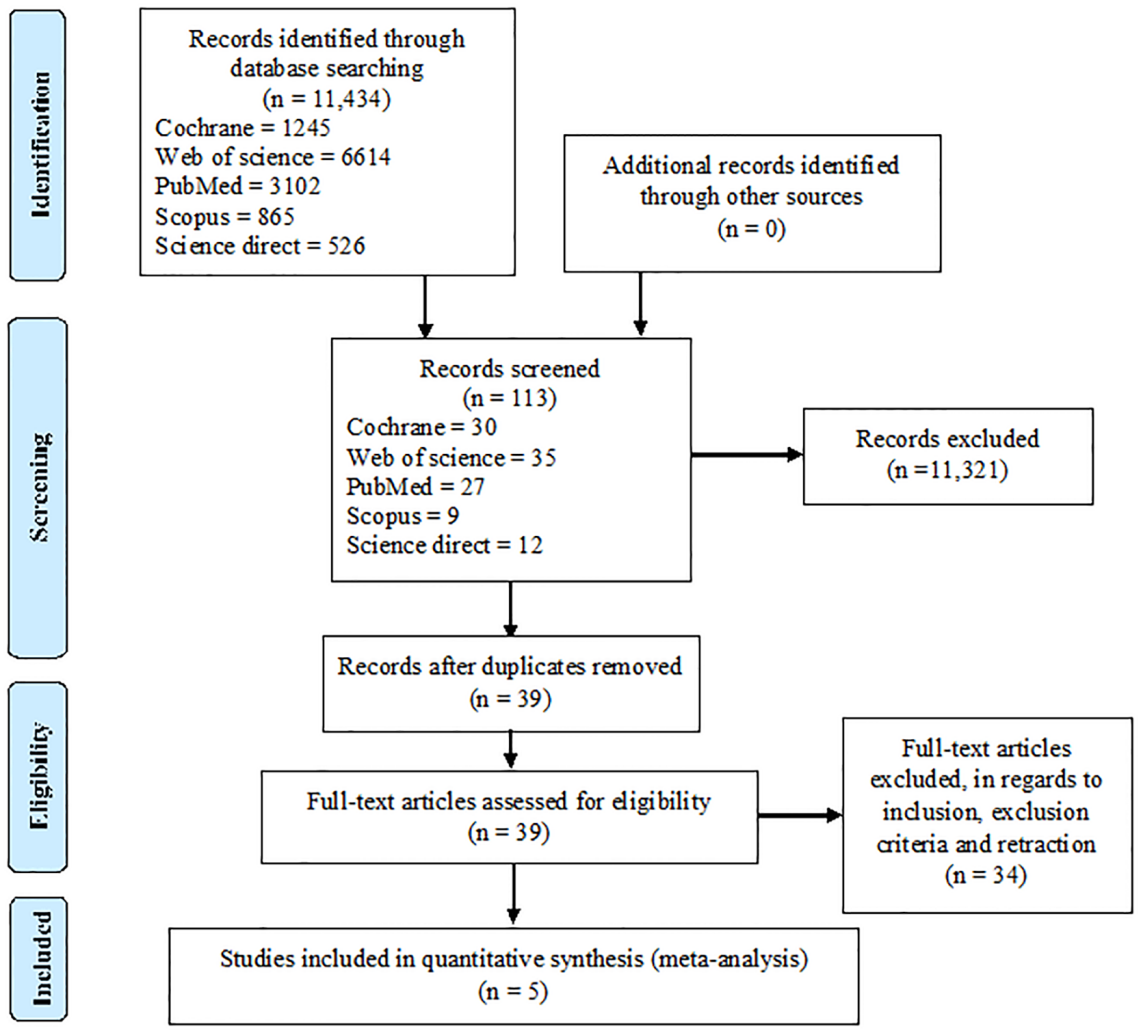
PRISMA flow chart outlining the literature search and the study selection process.

Our search identified 11,434 articles. Detailed characteristics of the included studies are given in S2 File. A PRISMA flow chart outlining the literature search and study selection process is given in Fig 1.

### Quality assessment

The quality assessment showed that four studies were of high quality and the remaining one of moderate quality. Agreement on the quality assessment between the observers ranged from 95 to 100% (S2 File). Other details of the quality of the studies are shown in S2 File.

### Description of the included studies

The characteristics of the included studies and participants are given in S2 File.

### Relationship between changes in MAP and ABPI and walking ability

**Fig 2:**
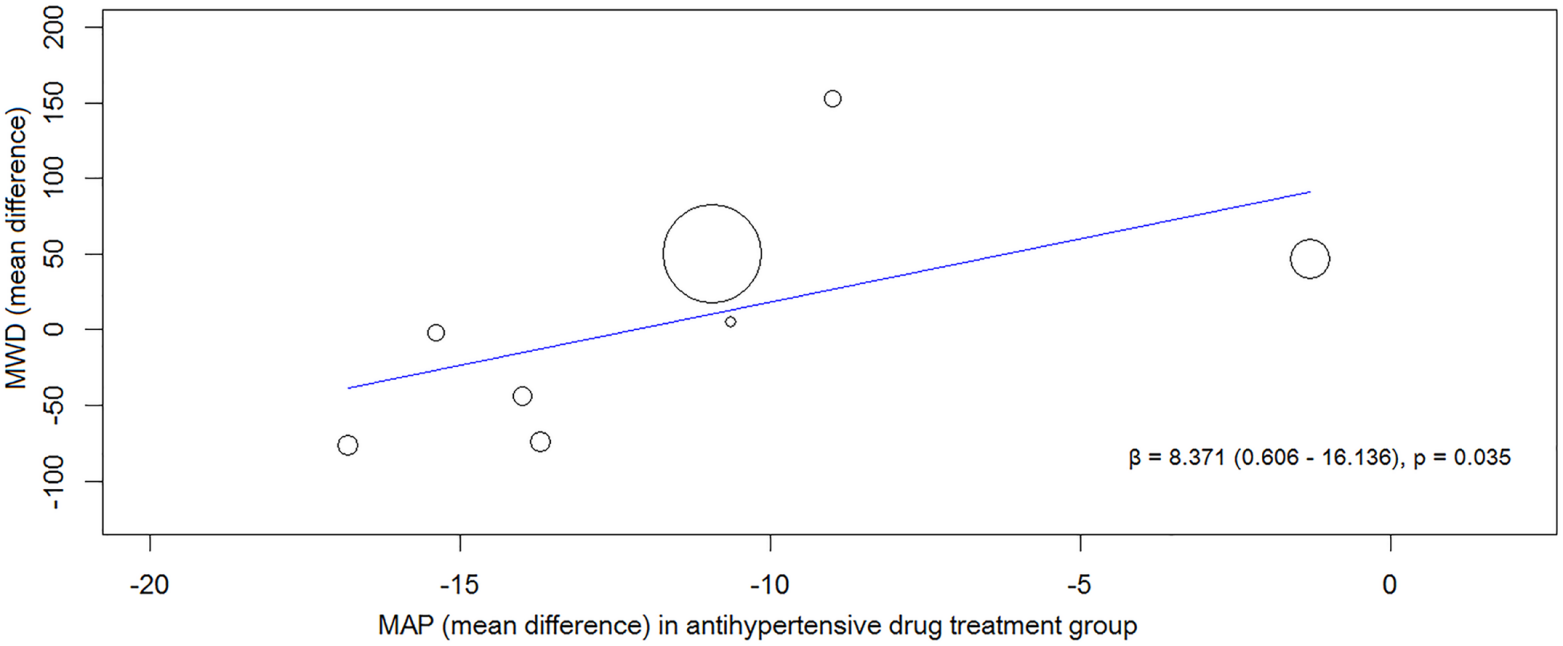
Results of the meta-regression analysis showing the association between reduction in blood pressure and improvement in MWD after being randomised to an anti-hypertensive drug. Fig 2 shows the association between reducing MAP and improving MWD in all the included trials. Abbreviations: β–regression coefficient, MAP—mean arterial pressure and MWD—maximum walking distance.

A meta-regression analysis was carried out to analyse whether the effect of the anti-hypertensives on MAP was associated with the changes in walking distance and ABPI recorded over the course of the trials. The change in MAP and walking distance after treatment with each anti-hypertensive drug was obtained by considering the reported parameters following a ‘washout period’ as baseline for the whole cohort. In total, 8 and 7 groups were used to evaluate the association between reduction in MAP after drug treatment with MWD and PFWD respectively. The magnitude of reduction in MAP from baseline due to the anti-hypertensive drugs showed a significant positive correlation with improved MWD (β = 8.371, p = 0.035) (Fig 2) but not PFWD or ABPI (PFWD, β = 4.276, p = 0.195; ABPI, β = -0.004, p = 0.357).

### The effect of anti-hypertensives on ABPI, MWD and PFWD

**Fig 3:**
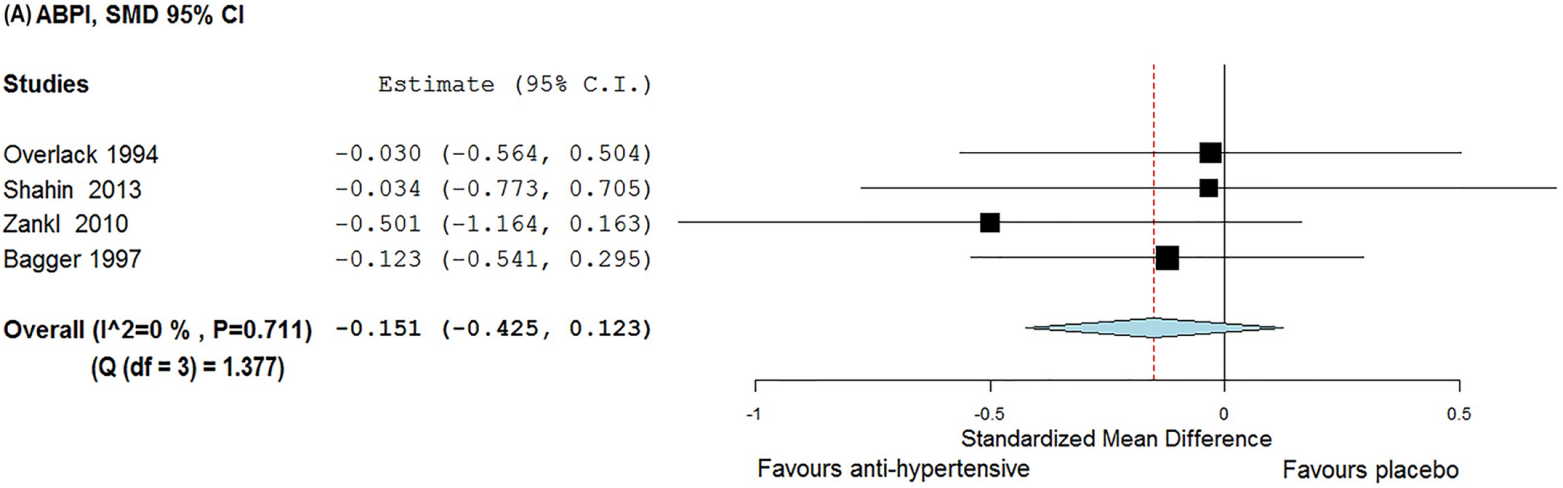
Forest plot illustrating the effect of anti-hypertensive medications on ABPI. The x-axis is the effect size expressed as SMD (standardised mean difference). The forest plot also details the 95% confidence interval. The dotted line represents the overall effect calculated in this meta-analysis. The heterogeneity estimated as I^2^ and Q is also provided. Abbreviation: ABPI—ankle brachial pressure index.

**Fig 4:**
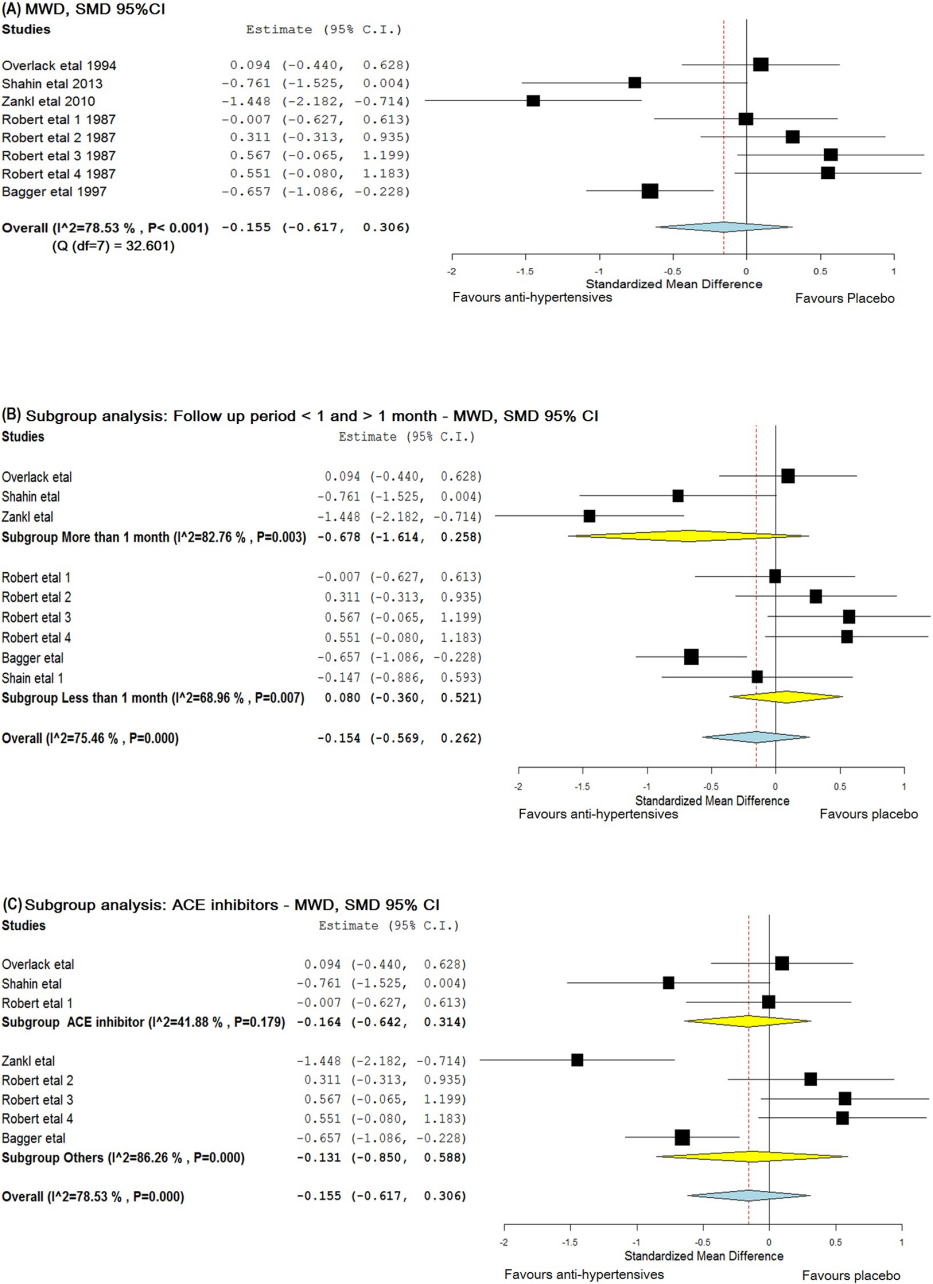
Forest plots illustrating the effect of anti-hypertensive medications on MWD. The x-axis is the effect size expressed as SMD (standardised mean difference). The forest plot also details the 95% confidence interval. The dotted line represents the overall effect calculated in this meta-analysis. The heterogeneity estimated as I^2^ and Q associated with each analysis is also provided. (A) The effect of anti-hypertensive medications on MWD, (B) Subgroup analysis including trials with follow up period greater than and less than 1 month, (C) Subgroup analysis including trials which used ACE inhibitors. Abbreviations: ACE—angiotensin converting enzyme and MWD—maximum walking distance.

**Fig 5:**
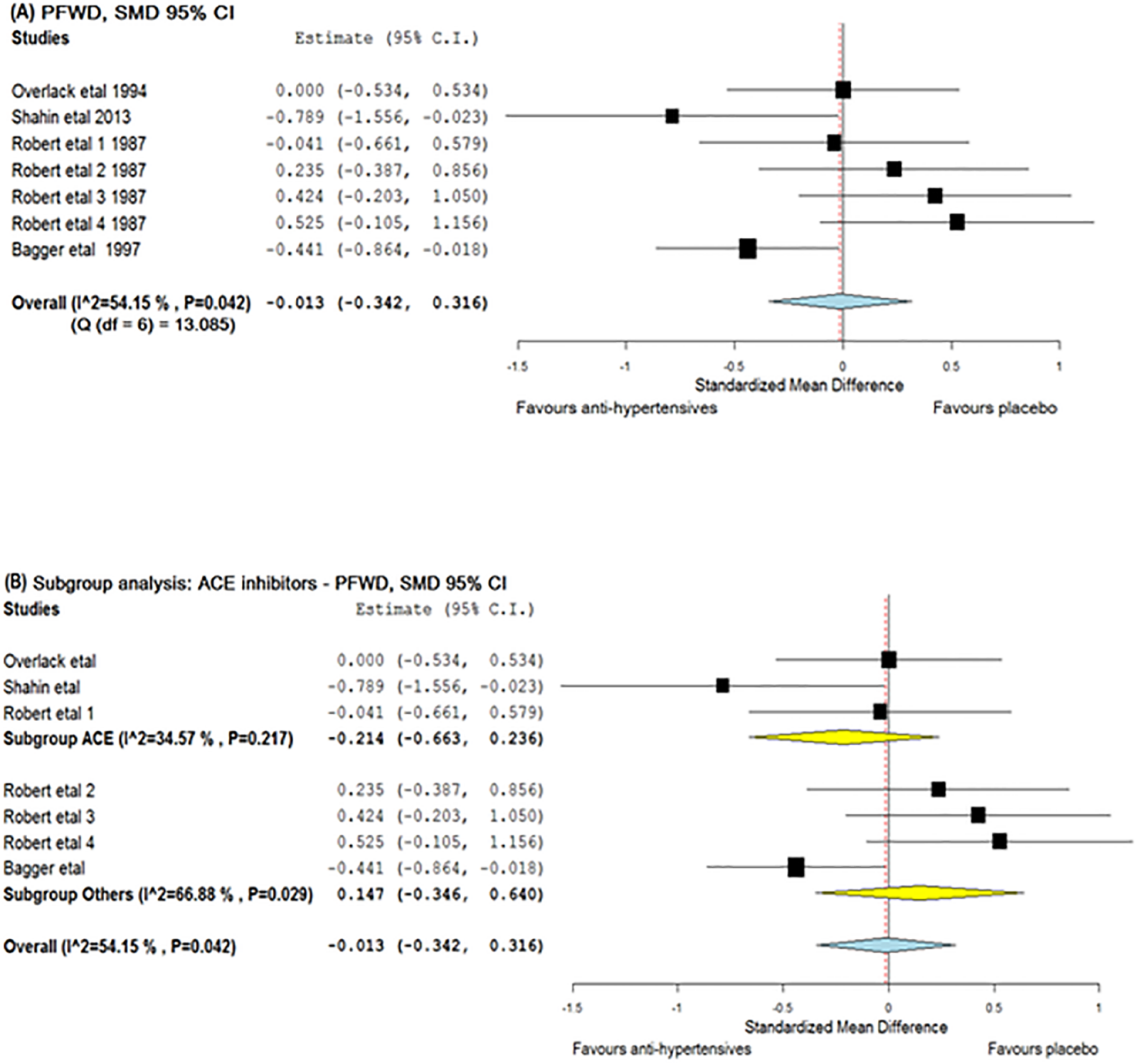
Forest plots illustrating the effect of anti-hypertensive medications on PFWD. The x-axis is the effect size expressed as SMD (standardised mean difference). The forest plot also details the 95% confidence interval. The dotted line represents the overall effect calculated in this meta-analysis. The heterogeneity estimated as I^2^ and Q associated with each analysis is also provided. (A) The effect of anti-hypertensive medications on PFWD, (B) Subgroup analysis including trials which included ACE inhibitors. Abbreviations: ACE—angiotensin converting enzyme and PFWD—pain free walking distance.

Meta-analyses were conducted to see if reported reductions in BP following antihypertensive therapy led to clinically relevant improvements in ABPI, MWD or PFWD. Studies by Robert *et al*. and Bagger *et al*. were given a baseline—post-intervention correlation value of 0.5 for this analysis. Robert *et al*. assessed 4 anti-hypertensive drugs and placebo in a crossover design. Hence for this meta-analysis, each treatment period was considered as a separate trial. Accordingly, a total of 8 and 7 groups were used to assess the effect of anti-hypertensives on MWD and PFWD respectively (S1 Table). The meta-analysis suggested that anti-hypertensives had no significant effect on ABPI [SMD = -0.151 (95% CI, -0.425–0.123)], MWD [SMD = -0.155 (95% CI, -0.617–0.306)] or PFWD [d = − 0.013 (95% CI, -0.342–0.316)] (Figs 3–5). The findings for MWD were similar in sub-group analyses that assessed treatment periods of > 1 or < 1 month (Fig 4B).

A sub-analysis was performed to determine whether ACE inhibitors had an effect on leg ischemia[18, 24, 38]. These studies had intervention period of 4, 6 and 24 weeks. No significant effect was found on either MWD (SMD = − 0.164, 95% CI -0.642–0.314) or PFWD (SMD = -0.214, 95% CI -0.663–0.236) (Figs 4C and 5B). Due to a lack of available studies, sub-analyses could not be performed for other drug classes.

### Assessment of publication bias

Eggers’s test and the funnel plots (S3 File) suggested no publication bias. The intercepts of the funnel plots assessing publication bias in trials included to analyse the effect of anti-hypertensive medications on ABPI, MWD and PFWD were -0.825 (-8.779–7.129, p = 0.699), 0.388 (-11.038–11.814, p = 0.936) and -1.205 (-14.505–12.095, p = 0.825) respectively.

## Discussion

The use and choice of anti-hypertensive medications in PAD patients has been debated for a long time and one of the main concerns have been that anti-hypertensive drugs may worsen leg ischemia. The main finding of this meta-analysis was that anti-hypertensive therapy did not affect leg ischemia as assessed by ABPI, MWD and PFWD. A benefit of anti-hypertensive medications on leg ischemia was suggested by the meta-regression since there was a significant positive correlation between degree of reduction in BP and improvement in MWD.

The RCTs included in this meta-analysis had many limitations. Notably, there was no washout period between treatment periods in the crossover trial by Robert *et al*.[18]. Hence significant carryover effects may have been present which may have masked the true effect of anti-hypertensive drugs tested. Furthermore, a follow-up period of at least 6 months is recommended in studies of IC to ensure that adequate time is available to demonstrate effects on the leg[39]. The trials by Bagger *et al*., Robert *et al*. and Overlack *et al*. had a follow-up period of two, four and six weeks respectively, which were lower than advocated[18, 23, 38]. Three trials included in this meta-analysis reported improvement in walking distance after anti-hypertensive drug treatment[23–25]. The treatment period for these trials were 52, 24 and 2 weeks, suggesting that longer duration of treatment with anti-hypertensives may be required to demonstrate their efficacy[39]. Only 2 trials achieved a SBP target according to current guidelines of ≤ 140 mmHg [24, 25]. Attaining BP goals may have improved the outcomes. The blinding details of the trial authored by Zankl *et al*. were unclear[25, 40]. The research was a single blinded study, however the paper did not specify whether the investigators or the patients were blinded. The risk of bias is significant when adequate blinding is not used[40]. RCTs without appropriate blinding have been demonstrated to show a larger treatment effect[41].

The overall lack of effect of anti-hypertensive medications on measures of leg ischemia reported in this meta-analysis can be attributed to many reasons. Firstly, there were few published trials that met the entry criteria and the majority of these trials included small number of patients. Secondly, there are potential methodological issues in combining crossover trials and parallel studies in a meta-analysis. The correlation co-efficient between baseline and follow-up scores in the included crossover trials were also not available hence a conservative estimate value of 0.5 was used[42]. Furthermore, the follow-up period was different in all the trials ranging from 2 to 52 weeks. Different trials also used different anti-hypertensive medications. The current meta-analysis was performed by pooling all trials together regardless of their anti-hypertensive class. In addition, the inclusion and exclusion criteria in the included RCTs were different as a result of which the baseline characteristics of the participants were noticeably different. The crossover trials excluded patients with concomitant coronary heart disease and diabetes mellitus[18, 23], whereas all the parallel trials included patients with these risk factors[24, 25, 38]. Therefore, the degree of heterogeneity between studies both in terms of the design and medications used was relatively high. Moreover, for analysis, data had to be represented as mean ± SD, hence several mathematical manipulations were needed to transform data into this format.

Despite these limitations, the current meta-analysis suggests that anti-hypertensive treatments do not worsen leg ischemia and may possibly improve MWD. This is important to confirm since lowering BP in patients with PAD has significant potential to reduce cardiovascular events. Current guidelines recommend the reduction of BP to ≤ 140/90 in PAD patients[16, 17] but a recent trial conducted by the SPRINT research group showed that a target of SBP ≤ 120 mmHg among patients at high risk of cardiovascular events, including PAD patients, resulted in substantially lower rates of cardiovascular events and associated mortality[43]. It is therefore likely that updated guidelines may advise lowering SBP in high risk patients to ≤ 120 mmHg.

The findings of one of our sub-analyses, that ACE inhibitors did not influence leg outcomes is in apparent contradiction to a previous meta-analysis which reported that ACE inhibitors improved walking ability in patients with IC[44]. It should be noted that the prior meta-analysis included two trials which have been subsequently retracted[45, 46] and positive data from these studies may have contributed to this difference in findings. However the results of this meta-analysis is comparable to an older meta-analysis which concluded that ACE inhibitors did not have any significant effect on walking distance and ABPI[47].

In conclusion, this meta-analysis suggests that reducing BP does not worsen leg ischemia in patients with IC. The role played by anti-hypertensives in improving outcomes is controversial and lacks sufficient evidence and has been clouded by retraction of two trials that reported positive outcomes[45, 46]. Large multicenter trials with prolonged anti-hypertensive treatment periods are required to clarify the effect of anti-hypertensives on symptoms of IC. However, designing such trials is difficult due to the convincing data suggesting that patients at high risk of cardiovascular events, such as PAD patients, should be on anti-hypertensive medications to reduce the incidence of such events[48, 49].

## Supporting information

S1 File Patients and methods. (DOCX)

S2 File Additional results. (DOCX)

S3 File Funnel plots and Egger’s test assessing publications bias. (DOCX)

S4 File PRISMA checklist. (DOC)

S1 Table Blood pressure and measures of leg ischemia reported in studies included in this meta-analysis. Results are expressed as mean ± SD. The mathematical approach taken for obtaining these data is given in S2 Table. Mean arterial pressure was defined from the following formula: MAP = [(2*DBP) + SBP] / 3. Different anti-hypertensives were used in the trials: Overlack trial—perindopril, Shahin *et al*.–ramipril, Zankl *et al*.–telmisartan, Bagger *et al*.–verapamil, Robert *et al*.–captopril, atenolol, labetolol and pindolol. Abbreviations: DBP- diastolic blood pressure, MAP—mean arterial pressure, PI—post-intervention and SBP—systolic blood pressure.
(DOCX)

S2 Table Calculations involved in the included trials. (DOCX)
