# Supplementary material for: Effect of blood pressure lowering medications on leg ischemia in peripheral artery disease patients: A meta-analysis of randomised controlled trials
Source: PLoS One. 2017 Jun 2;12(6):e0178713. doi: 10.1371/journal.pone.0178713 (PMC5456103; doi:10.1371/journal.pone.0178713)
Supplement: S1 File — (DOCX) [file pone.0178713.s001.docx]

**Supplementary File 1**

**Patients and Methods**

**Search strategy**

The search was conducted using the Cochrane Central Register of Controlled Trials (CENTRAL), PubMed, Web of Knowledge, Science Direct and Scopus databases on May 2016 using the following search terms: ‘peripheral artery disease’ OR ‘peripheral arterial disease’ OR ‘peripheral vascular disease’ OR ‘arterial occlusive disease’ OR ‘intermittent claudication’ OR ‘PAD’ OR ‘PVD’ OR ‘POAD’ AND ‘anti-hypertensive agents’ OR ‘anti-hypertensive drugs’ OR ‘anti-hypertensives’. Further searches were performed using all anti-hypertensive classes and individual anti-hypertensive drug names as mentioned in the below table.

**Table 1: Additional search terms used in this meta-analysis.**

| Anti-hypertensive drug classes | ‘adrenergic alpha-antagonists’ or ‘adrenergic beta-antagonists’ or ‘angiotensin-converting enzyme inhibitors’ or ‘diuretics’ or ‘ganglionic blockers’ or ‘vasodilator agents’ or ‘anti-arrhythmic agents’ or ‘vasodilator agents’ OR ‘calcium channel blocker’ OR |
| --- | --- |
| Anti-hypertensive drugs | doxazosin’ OR ‘prazosin’ OR ‘terazosin’ OR ‘bumetanide’ OR ‘ethacrynic acid’ OR ‘furosemide’ OR ‘torsemide’ OR ‘hydrochlorthiazide’ OR ‘indapamide’ OR ‘metolazone’ OR ‘amiloride’ OR ‘spironolactone’ OR ‘amlodipine’ OR ‘cilnidipine’ OR ‘felodipine’ OR ‘isradipine’ OR ‘lercanidipine’ OR ‘levamlodipine’ OR ‘nicardipine’ OR ‘nifedipine’ OR ‘nimodipine’ OR ‘nitrendipine’ OR ‘diltiazem’ OR ‘verapamil’ OR ‘captopril’ OR ‘enalapril’ OR ‘fosinopril’ OR ‘lisinopril’ OR ‘perindopril’ OR ‘quinapril’ OR ‘ramipril’ OR ‘trandolapril’ OR ‘benazepril’ OR ‘candesartan’ OR ‘eprosartan’ OR ‘irbesartan’ OR ‘losartan’ OR ‘olmesartan’ OR ‘telmisartan’ OR ‘valsartan’ OR ‘doxazosin’ OR ‘phentolamine’ OR ‘indoramin’ OR ‘phenoxybenzamine’ OR ‘prazosin’ OR ‘terazosin’ OR ‘tolazoline’ OR ‘clonidine’ OR ‘guanabenz’ OR ‘guanfacine’ OR ‘methyldopa’ OR ‘moxonidine’ OR ‘eplerenone’ OR ‘spironolactone’ OR ‘isosorbide’. |

The searches were limited to clinical trials performed in humans and published in English. The titles and abstracts of the studies identified were examined by the first author (DT) to identify relevant articles. The full text of these articles were then evaluated in relation to the inclusion and exclusion criteria.

**Study selection**

The studies included had either a placebo controlled parallel or crossover study design with clear diagnostic criteria for PAD (ABPI < 0.9, imaging evidence of PAD or clinical findings consistent with PAD)[1] and included patients with concomitant hypertension and IC. A previous systematic review suggested that 50% of maximum BP lowering is achieved within the first two weeks of treatment[2]. Therefore a minimum anti-hypertensive treatment period of 2 weeks was required. Baseline and follow-up mean arterial pressure (MAP) and ABPI, MWD or PFWD values had to be reported. Studies that included patients with rest pain, leg ulcers or gangrene were excluded.

**Data extraction**

Two authors (DT and BD) extracted data independently using a predefined data extraction table (S2 file and S1 table).The following data were extracted: study design; patient characteristics (including age, proportion of males and cardiovascular risk factors); baseline and follow-up values of MAP, ABPI, PFWD and MWD. Missing data were sought by contacting the corresponding author of the publication concerned. Extrapolation of data from figures were achieved using Adobe Photoshop CC (2015) in studies that did not report numerical results. Any data discrepancies were resolved through discussion (with a third author SK where necessary). The Overlack trial[3] did not include results for MAP but had mean values of systolic and diastolic blood pressure from which MAP was calculated with the help of the formula MAP = 2 * diastolic blood pressure (DBP) + systolic blood pressure (SBP) / 3[4]. The discrete standard errors (SE) of DBP and SBP were used to derive the standard error of mean (SEM) of MAP (S2 table). In studies reporting median ± interquartile range (IQR) or mean ± SEM or median ± confidence interval (CI), data were converted to mean ± standard deviation (SD) using standard formulas published in the Cochrane handbook for systematic reviews of intervention[5] and by Wan *et al*[6]. Shahin *et al* reported the baseline values and adjusted mean change ± SEM after the intervention period[7]. Statistical analyses were performed on these data to calculate approximate post-intervention values as detailed in S2 table.

**Study quality assessment**

A quality assessment tool was formulated using components from the following validated tools: a) The 25 items CONSORT 2010 checklist of information to be included when reporting a randomised trial; and b) The Cochrane collaboration tool for assessing risk of bias [5,8]. The quality assessment tool was modified to include questions that were specific to PAD, hypertension and walking ability research (S2 file). Quality assessment of the included studies was conducted initially by the first author (DT) followed by two other authors (BD and SK). The latter authors were blinded to names, affiliations, corresponding addresses and details of the publishing journal. A significant difference in opinion was considered when a difference of 2 or more points in the quality scoring was present. Any differences in opinion were discussed in a consensus meeting in order to reach a resolution. One of the included questions in the tool was only relevant for parallel studies, hence the total score of quality assessment was different for parallel and crossover studies. Mean quality assessment scores were calculated by averaging scores of all three assessors. Studies were regarded as high, moderate, or low quality if the average quality assessment scores were ≥ 75%, 50 - 75%, or ≤ 50%, respectively.

**Quantitative data synthesis**

The first author (DT) performed statistical analysis of data using the Meta-Analyst software (version beta 3.13)[9]. Both parallel and crossover trials were included in this meta-analysis. The Meta-Analyst software allows us to estimate the overall effect size (expressed as standardised mean difference (SMD)) of anti-hypertensive medications by using the original mean and respective SD values at baseline and post-intervention of both groups (placebo and anti-hypertensive drug)[10]. Since crossover trials were included in this meta-analysis, the correlation between baseline and follow-up scores were required from the literature for analysis. Neither Bagger *et al* nor Robert *et al* [11,12] reported the expected within – patient correlation. Hence a correlation of 0.5 was assigned to the crossover trials which is considered as a conservative estimate when using the change score according to Follmann *et al* and Rongwei *et al* [13,14].

A random effects model was used in this meta-analysis anticipating that heterogeneity between studies would be likely. The heterogeneity among the studies was assessed using the I^2^ test. I^2^ values corresponding to 25%, 50% and 75% were considered as low, moderate and high heterogeneity respectively[15]. Sensitivity analyses were performed using the leave one out method to assess the impact of each study on the combined effect size. Meta-regression was also carried out to analyse the association between the degree of changes in MAP following anti-hypertensive therapy and changes in ABPI, MWD and PFWD. Mean difference in MAP between the baseline and the post-intervention phase was added as a covariate to the outcomes of interest (ABPI, MWD and PFWD). Sub-analyses were carried out to examine the effect of different anti-hypertensive medications and different treatment periods if at least three original studies assessing the same class of drug were available to estimate the overall effect size. Publication bias was assessed using funnel plots corresponding to each result. For all analyses p values <0.05 were considered to be statistically significant.

**References**

1. Au TB, Golledge J, Walker PJ, Haigh K, Nelson M. Peripheral arterial disease: diagnosis and management in general practice. Aust Fam Physician. 2013;42(6):397.
2. Lasserson DS, Buclin T, Glasziou P. How quickly should we titrate antihypertensive medication? Systematic review modelling blood pressure response from trial data. Heart. 2011;97(21):1771-5.
3. Overlack A, Adamczak M, Bachmann W, Bonner G, Bretzel RG, Derichs R, et al. ACE-inhibition with perindopril in essential hypertensive patients with concomitant diseases. The Perindopril Therapeutic Safety Collaborative Research Group. Am J Med. 1994;97(2):126-34.
4. Cywinski J, Tardieu B. The essentials in pressure monitoring Blood and other body fluids. Ann Intern Med. 1981;95(3):399-400.
5. Higgins JPT, Green S (editors). Cochrane handbook for systematic reviews of interventions version 5.1. 0 [updated March 2011]. The Cochrane Collaboration, 2011. Available from [www.cochrane-handbook.org](http://www.cochrane-handbook.org).
6. Wan X, Wang W, Liu J, Tong T. Estimating the sample mean and standard deviation from the sample size, median, range and/or interquartile range. BMC Med Res Methodol. 2014;14(1):135.
7. Shahin Y, Cockcroft JR, Chetter IC. Randomized clinical trial of angiotensin-converting enzyme inhibitor, ramipril, in patients with intermittent claudication. Br J Surg. 2013;100(9):1154-63.
8. Schulz KF, Altman DG, Moher D. CONSORT 2010 statement: updated guidelines for reporting parallel group randomised trials. BMJ. 2010;340:c332.
9. Wallace BC, Schmid CH, Lau J, Trikalinos TA. Meta-Analyst: software for meta-analysis of binary, continuous and diagnostic data. BMC Med Res Methodol. 2009;9(1):80.
10. Rosenthal JA. Qualitative descriptors of strength of association and effect size. J Soc Serv Res. 1996;21(4):37-59.
11. Bagger JP, Helligsoe P, Randsbaek F, Kimose HH, Jensen BS. Effect of verapamil in intermittent claudication A randomized, double-blind, placebo-controlled, cross-over study after individual dose-response assessment. Circulation. 1997;95(2):411-4.
12. Roberts DH, Tsao Y, McLoughlin GA, Breckenridge A. Placebo-controlled comparison of captopril, atenolol, labetalol, and pindolol in hypertension complicated by intermittent claudication. Lancet. 1987;2(8560):650-3.
13. Follmann D, Elliott P, Suh I, Cutler J. Variance imputation for overviews of clinical trials with continuous response. J Clin Epidemiol. 1992;45(7):769-73.
14. Rongwei Fu, Benjamin W.V et al. Handling continuous outcomes in quantitative synthesis. Methods guide for effectiveness and comparative effectiveness reviews. Rockville, MD: Agency for Healthcare Research and Quality: 2014. p. 285 – 311.
15. Higgins JP, Thompson SG, Deeks JJ, Altman DG. Measuring inconsistency in meta-analyses. BMJ. 2003;327(7414):557-60.
