# Supplementary material for: Effect of blood pressure lowering medications on leg ischemia in peripheral artery disease patients: A meta-analysis of randomised controlled trials
Source: PLoS One. 2017 Jun 2;12(6):e0178713. doi: 10.1371/journal.pone.0178713 (PMC5456103; doi:10.1371/journal.pone.0178713)
Supplement: S2 File — (DOCX) [file pone.0178713.s002.docx]

**Supplementary File 2**

**Results**

**Characteristics of the included studies**

Our search identified 11,434 articles. The review of their title and abstract led to selection of 113 articles for further assessment. After removal of duplicates (n = 74), 39 full text publications were reviewed. Thirty four of these publications were excluded as they did not fulfill the inclusion criteria (Fig 1). Most of the articles were excluded due to one of the following reasons: i) they did not report baseline values of MAP, ABPI, MWD or PFWD; ii) the study was not placebo-controlled; or iii) the investigation did not report any outcomes of interest. Two studies which fulfilled the inclusion criteria were excluded as they were subsequently retracted by the authors[1, 2]. Out of the remaining 5 studies, 4 reported ABPI[3-6] and PFWD[3, 4, 6, 7], whereas all 5 reported MWD[3-7]. The 5 studies that reported ABPI, MWD or PFWD and MAP at baseline and post-intervention after placebo and drug administration were included in this meta-analysis.

**Quality assessment**

The quality assessment showed that four studies were of high quality and the remaining one of moderate quality. Agreement on the quality assessment between the observers ranged from 95 to 100% (Table 1). All the studies defined aims and study design clearly but three failed to mention the trial setting[4, 5, 7] and two did not provide information on the randomisation method used[3, 5]. One of the included trial was single blinded and the details of blinding were unclear[5]. The exclusion criteria for participants was not mentioned in one trial[3]. There was no documentation of baseline demographics in one trial[6] and clinical characteristics of participants in three trials[3, 6, 7]. The clinical characteristics were similar in the two patient groups in two parallel trials[4, 5] whereas the third trial did not report this aspect[3]. Two studies only presented results graphically [4, 7]. All the trials reported the number of drop outs and every outcome of interest was detailed in the results.

**Table 1: Assessment of methodological quality of studies**

| No | Study | Overlack *et al* | | | Robert *et al* | | | Shahin *et al* | | | Bagger *et al* | | | Zankl *et al* | | |
| --- | --- | --- | --- | --- | --- | --- | --- | --- | --- | --- | --- | --- | --- | --- | --- | --- |
|  | Questions | DT | SK | BD | DT | SK | BD | DT | SK | BD | DT | SK | BD | DT | SK | BD |
| 1 | Was the aim of the study reported? | Y | Y | Y | Y | Y | Y | Y | Y | Y | Y | Y | Y | Y | Y | Y |
| 2 | Was the trial design reported (parallel or crossover) including allocation ratio (if parallel)? | Y | Y | Y | Y | Y | Y | Y | Y | Y | Y | Y | Y | Y | Y | Y |
| 3 | Was the study setting (hospital, clinic or nursing home) defined? | Y | Y | Y | N | N | U | N | N | N | Y | Y | Y | N | N | N |
| 4 | Were all the participants recruited from the same setting? | N | N | N | Y | Y | Y | Y | Y | Y | Y | Y | Y | Y | Y | Y |
| 5 | Was the method of randomisation (simple, block, stratified or covariate) reported? | N | N | U | Y | Y | Y | Y | Y | Y | Y | Y | N | N | U | N |
| 6 | Were the study participants blinded? | Y | Y | Y | Y | Y | Y | Y | Y | Y | Y | Y | Y | Y | Y | Y |
| 7 | Were the other personnel (care providers, those assessing outcomes) involved in the trial blinded? | Y | Y | Y | Y | Y | Y | Y | Y | Y | Y | Y | Y | N | N | N |
| 8 | Did the study state clear inclusion criteria for participants? | Y | Y | Y | Y | Y | Y | Y | Y | Y | Y | Y | Y | Y | Y | Y |
| 9 | Did the study state clear exclusion criteria for participants? | N | N | N | Y | Y | Y | Y | Y | Y | Y | Y | Y | Y | Y | Y |
| 10 | Was the diagnosis of PAD defined appropriately? (A diagnosis by either of the following: ABPI <0.9, Angiography or other clinical imaging techniques or physical examination) | Y | Y | Y | Y | Y | Y | Y | Y | Y | Y | Y | Y | U | Y | U |
| 11 | Were participants with uncontrolled hypertension excluded from the study (BP > 160/90)? | Y | Y | Y | Y | Y | Y | Y | Y | Y | Y | Y | Y | Y | Y | Y |
| 12 | Did the study clearly report the interventions for each group with sufficient details (how and when they were administered)? | Y | Y | Y | Y | Y | Y | Y | Y | Y | Y | Y | Y | Y | Y | Y |
| 13 | Did the study define primary and secondary outcomes (how and when they were assessed)? | Y | Y | Y | Y | Y | Y | Y | Y | Y | Y | Y | Y | Y | Y | Y |
| 14 | Did the study report drop out numbers? | Y | Y | Y | Y | Y | Y | Y | Y | Y | Y | Y | Y | Y | Y | Y |
| 15 | Did the study detail the baseline demographics (age, sex) of the participants? | Y | Y | Y | Y | Y | Y | Y | Y | Y | N | N | N | Y | Y | Y |
| 16 | Did the study detail the clinical characteristics (cardiovascular risk factors, ABPI) of the participants? | N | N | N | N | N | N | Y | Y | Y | N | N | N | Y | Y | Y |
| 17 | Were the following clinical characteristics similar between the participant groups  (In parallel studies)? | | | | | | | | | | | | | | | |
|  | 1. Smoking | N | N | N | - | - | - | Y | Y | Y | - | - | - | Y | Y | Y |
|  | 1. Diabetes mellitus | N | N | N | - | - | - | Y | Y | Y | - | - | - | Y | Y | Y |
|  | 1. Dyslipidaemia | N | N | N | - | - | - | Y | Y | Y | - | - | - | Y | Y | Y |
| 18 | Was the walking distance calculated with the help of standard tests (treadmill or 6 minute walk test)? | Y | Y | Y | Y | Y | Y | Y | Y | Y | Y | Y | Y | Y | Y | Y |
| 19 | For the outcomes of interest (systolic blood pressure, maximum walking distance and ankle brachial pressure index), were the results stated clearly? | Y | Y | Y | N | N | N | N | N | N | Y | Y | Y | Y | Y | Y |
| 20 | Were the outcomes stated with a p value? | Y | Y | Y | Y | Y | Y | Y | Y | Y | Y | Y | Y | Y | Y | Y |
| 21 | Were all the pre-specified outcomes (outcomes mentioned in methods) reported? | Y | Y | Y | Y | Y | Y | Y | Y | Y | Y | Y | Y | Y | Y | Y |
| Individual scores (Y) | | 16/23 | 16 | 16 | 17/20 | 17 | 17 | 21/23 | 21 | 21 | 18/20 | 18 | 17 | 19/23 | 20 | 19 |
| Concentrations of agreement (%) | | 100 | | | 100 | | | 100 | | | 95 | | | 95.6 | | |
| Averaged score (%) | | 69.6 | | | 85 | | | 91.3 | | | 88.3 | | | 84 | | |

Abbreviations: ABPI – ankle brachial pressure index, BP- blood pressure, PAD- peripheral artery disease, DT- Diana Thomas Manapurathe, SK- Smriti Murali Krishna, BD- Brittany Dewdney, Y- yes, N- no, U-unclear. This modified quality assessment questionnaire was developed to assess the quality of the included trials.

**Description of the included studies**

The characteristics of the included studies and participants are given in table 2. Two of the included studies had a crossover study design[6, 7] and the other three were parallel trials[3-5]. The mean age of patients ranged from 56 to 65 years and all studies had a high proportion of men. All trials used different anti-hypertensive drugs including the angiotensin converting enzyme (ACE) inhibitors ramipril, perindopril and captopril, the angiotensin receptor blocker (ARB) telmisartan, the calcium channel blocker verapamil, and the β blockers atenolol, labetolol and pindolol. One of the placebo controlled crossover trial used four anti-hypertensive drugs including captopril, atenolol, labetalol and pindolol. Each treatment period in this trial lasted for only one month[7].

A total of 180 patients receiving anti-hypertensives and 127 patients receiving placebo were investigated across all five studies. The intervention period was different in all five trials. In the studies by Zankl *et al,* Shahin *et al* and Overlack *et al* the intervention periods were 24, 52 and 6 weeks, respectively[3-5]. The intervention period in the other 2 included studies were less than a month (Bagger *et al*: 2 weeks and Robert *et al*: 4 weeks[6, 7]).

The two crossover trials excluded patients with coronary heart disease (CHD) and diabetes mellitus (DM)[6, 7], whereas in one parallel trial participants had a high prevalence of CHD (94.4% in the anti-hypertensive and 88.9% in placebo group)[5]. More than 25% of the participants in two trials had DM[4, 5]. The crossover trials did not provide any data on the proportion of patients with hyperlipidemia, nor patients’ mean body mass index (BMI)[6, 7]. The study by Overlack *et al* was a trial carried out to study the effect of perindopril on several diseases including PAD. Patient demographic and clinical characteristics data were only provided for the entire population, hence no separate data were available regarding the subset of PAD participants[3]. The blood pressure and PAD severity measurements were recorded separately for the respective subset of patients. The prevalence of current smoking was reported in all trials and varied from 22 – 85%.

Current treatment guidelines recommend that the BP in PAD patients should be controlled to ≤ 140/90. The mean SBP after anti-hypertensive treatment in the Overlack trial was above 140 mmHg[3]. Robert *et al* reported only mean MAP which was also higher than recommended levels (MAP for BP 140/90 = 107 mmHg) after administration of anti-hypertensives[7]. Bagger *et al* achieved a mean MAP of 102.5 which was within the advocated range[6] and the anti-hypertensive medication treated populations of two trials (Shahin *et al* and Zankl *et al*) achieved a mean SBP target ≤ 140 mmHg[4, 5].

**Table 2: Characteristics of the randomised control studies included in this meta-analysis**

| **Study** | **Year** | **Country** | **Design** | **Blinding** | **n** | **Drug used** | **Dose/day**  **(mg)** | **Drug (n)** | **Placebo (n)** | **Follow-up** | **Outcomes assessed** | **Drug/placebo** | **Age** | **Male %** | **Clinical characteristics** | | | | |
| --- | --- | --- | --- | --- | --- | --- | --- | --- | --- | --- | --- | --- | --- | --- | --- | --- | --- | --- | --- |
|  |  |  |  |  |  |  |  |  |  |  |  |  |  |  | **BMI** | **Hyperlipidemia** | **CAD** | **DM** | **Current smokers %** |
| Robert *et al* | 1987 | United Kingdom | Cross  over | Double | 23 | Captopril | 50 | 20 | 20 | 4 weeks | MAP, HR, CBF, PFWD and MWD | Drug/placebo | 58.0± 9.6 | 73.9 | - | - | Excluded | Excluded | 39.1 |
|  |  |  |  |  | 23 | Atenolol | 100 | 20 | 20 | 4 weeks |  |  |  |  |  |  |  |  |  |
|  |  |  |  |  | 23 | Labetolol | 400 | 20 | 20 | 4 weeks |  |  |  |  |  |  |  |  |  |
|  |  |  |  |  | 23 | Pindolol | 20 | 20 | 20 | 4 weeks |  |  |  |  |  |  |  |  |  |
| Overlack *et al* | 1994 | Germany | Parallel | Double | 54 | Perindopril | 4 | 26 | 28 | 6 weeks | SBP, DBP, HR, ABPI, PFWD and MWD | Drug | 59.3 ± 3.6 | 51.3 | - | - | - | - | - |
|  |  |  |  |  |  |  |  |  |  |  |  | placebo | 59.1 0± 3.7 | 56.1 | - | - | - | - | - |
| Bagger *et al* | 1997 | United Kingdom | Cross over | Double | 44 | Verapamil | 120 or 240 or 360 or 480 | 44 | 44 | 2 weeks | MAP, ABPI, PFWD, MWD, HR, PLT, SPLP | Drug/placebo | 59.0 ± 37.8 | - | - | - | Excluded | Excluded | 84.1 |
| Zankl *et al* | 2010 | Germany | Parallel | Single | 36 | Telmisartan | 40 or 80 | 18 | 18 | 52 weeks | SBP, DBP, MWD,MAP, FMV, ABPI, IMT, DRQOL | Drug | 56.0 ± 3.0 | 72.2 | 26.0 ± 0.8 | 100.0 | 94.4 | 27.8 | 44.4 |
|  |  |  |  |  |  |  |  |  |  |  |  | placebo | 62.2 ± 5.2 | 72.2 | 25.8 ± 0.8 | 94.4 | 88.9 | 27.8 | 22.2 |
| Shahin *et al* | 2013 | United Kingdom | Parallel | double | 33 | Ramipril | 5 mg : 2 weeks; 10mg 22 weeks | 12 | 17 | 24 weeks | SBP, DBP, MAP, ABPI, PFWD, HR, MWD, arterial stiffness, QOL and laboratory measurements. | Drug | 64.4 ± 8.2 | 78.5 | 28.1 0± 3.8 | 85.7 | 14.2 | 28.6 | 50.0 |
|  |  |  |  |  |  |  |  |  |  |  |  | Placebo | 64.7 ± 7.7 | 73.7 | 28.3 ± 4.2 | 94.7 | 10.5 | 36.8 | 47.4 |

Age is presented as mean ± SD, Sex and clinical characteristics presented in %. n: total participants at the start of the trial, drug (n) and placebo (n): the number of participants in each group after drop out. Abbreviations: ABPI – ankle – brachial pressure index, CBF – calf blood flow, DBP – diastolic blood pressure, DRQOL - Disease related quality of life, FMV – flow mediated vasodilatation, HR – heart rate, IMT - Intima- media thickness, MAP- mean arterial pressure, MWD – maximum walking distance, PFWD – pain free walking distance and PLT – peripheral leg temperature, QOL – quality of life, SBP – systolic blood pressure and SPLP – systolic peripheral leg pressure.

**References**

1. Ahimastos AA, Lawler A, Reid CM, Blombery PA, Kingwell BA. Brief communication: ramipril markedly improves walking ability in patients with peripheral arterial disease: a randomized trial. *Ann Intern Med.* 2006;144(9):660-4.
2. Ahimastos AA, Walker PJ, Askew C, Leicht A, Pappas E, Blombery P, et al. Effect of ramipril on walking times and quality of life among patients with peripheral artery disease and intermittent claudication: a randomized controlled trial. *JAMA*. 2013;309(5):453-60.
3. Overlack A, Adamczak M, Bachmann W, Bonner G, Bretzel RG, Derichs R, et al. ACE-inhibition with perindopril in essential hypertensive patients with concomitant diseases. The Perindopril Therapeutic Safety Collaborative Research Group. *Am J Med*. 1994;97(2):126-34.
4. Shahin Y, Cockcroft JR, Chetter IC. Randomized clinical trial of angiotensin-converting enzyme inhibitor, ramipril, in patients with intermittent claudication. *Br J Surg*. 2013;100(9):1154-63.
5. Zankl AR, Ivandic B, Andrassy M, Volz HC, Krumsdorf U, Blessing E, et al. Telmisartan improves absolute walking distance and endothelial function in patients with peripheral artery disease. *Clin Res Cardiol.* 2010;99(12):787-94.
6. Bagger JP, Helligsoe P, Randsbaek F, Kimose HH, Jensen BS. Effect of verapamil in intermittent claudication A randomized, double-blind, placebo-controlled, cross-over study after individual dose-response assessment. *Circulation*. 1997;95(2):411-4.
7. Roberts DH, Tsao Y, McLoughlin GA, Breckenridge A. Placebo-controlled comparison of captopril, atenolol, labetalol, and pindolol in hypertension complicated by intermittent claudication. *Lancet*. 1987;2(8560):650-3.
