# Supplementary material for: Effect of blood pressure lowering medications on leg ischemia in peripheral artery disease patients: A meta-analysis of randomised controlled trials
Source: PLoS One. 2017 Jun 2;12(6):e0178713. doi: 10.1371/journal.pone.0178713 (PMC5456103; doi:10.1371/journal.pone.0178713)
Supplement: S3 File — (DOCX) [file pone.0178713.s003.docx]

**Supplementary file 3**

**Funnel plots and Egger’s test assessing publications bias**

| 1. Funnel plot assessing publication bias: trials assessing ABPI.     Bias indicator  Egger: -0.82506 (-8.77929 – 7.12918) p = 0.69905 |
| --- |
| 1. Funnel plot assessing publication bias: trials assessing MWD     Bias indicator  Egger: 0.38805 (-11.03786 – 11.81396) p = 0.93647 |
| 1. Funnel plot assessing publication bias: trials assessing PFWD     Bias indicator  Egger: -1.20485 (-14.50506 – 12.09535) p = 0.82510 |
