# Supplementary material for: Effect of blood pressure lowering medications on leg ischemia in peripheral artery disease patients: A meta-analysis of randomised controlled trials
Source: PLoS One. 2017 Jun 2;12(6):e0178713. doi: 10.1371/journal.pone.0178713 (PMC5456103; doi:10.1371/journal.pone.0178713)
Supplement: S1 Table — Results are expressed as mean ± SD. The mathematical approach taken for obtaining these data is given in S2 Table. Mean arterial pressure was defined from the following formula: MAP = [(2*DBP) + SBP] / 3. Different anti-hypertensives were used in the trials: Overlack trial—perindopril, Shahin et al.–ramipril, Zankl et al.–telmisartan, Bagger et al.–verapamil, Robert et al.–captopril, atenolol, labetolol and pindolol. Abbreviations: DBP- diastolic blood pressure, MAP—mean arterial pressure, PI—post-intervention and SBP—systolic blood pressure. (DOCX) [file pone.0178713.s005.docx]

| **Study (drug)** | **Mean arterial pressure (MAP) (mmHg)** | | | | **Ankle brachial pressure index (ABPI)** | | | | **Maximum walking distance (MWD)(meters)** | | | | **Pain free walking distance (PFWD)(meters)** | | | |
| --- | --- | --- | --- | --- | --- | --- | --- | --- | --- | --- | --- | --- | --- | --- | --- | --- |
|  | **Placebo** | | **Drug** | | **Placebo** | | **drug** | | **Placebo** | | **drug** | | **Placebo** | | **drug** | |
|  | **Baseline** | **PI** | **Baseline** | **PI** | **Baseline** | **PI** | **Baseline** | **PI** | **Baseline** | **PI** | **Baseline** | **PI** | **Baseline** | **PI** | **Baseline** | **PI** |
| Overlack *et al* (perindopril) | 118.33 ± 9.22 | 114.76 ± 12.98 | 119.66 ± 13.50 | 109.03 ± 14.35 | 0.68 ± 0.21 | 0.75 ± 0.26 | 0.67 ± 0.20 | 0.75 ± 0.25 | 333.00 ± 227.47 | 369.00 ± 243.34 | 318.00 ± 229.50 | 323.00 ± 219.30 | 154.00 ± 58.19 | 165.00 ± 52.90 | 162.00 ± 76.49 | 173.00 ± 86.68 |
| Shahin *et al* (Ramipril) | 105.00 ± 12.37 | 107.00 ± 14.86 | 99.00 ± 10.40 | 90.00 ± 12.48 | 0.66 ± 0.16 | 0.68 ± 0.24 | 0·59 ± 0.19 | 0.62 ± 0.20 | 142.66 ± 113.80 | 165.57 ± 138.38 | 153.33 ± 85.40 | 306.43 ± 117.92 | 96.33 ± 105.73 | 116.53 ± 139.03 | 81.00 ± 54.73 | 225.09 ± 98.75 |
| Zankl *et al* (telmisartan) | 114.26 ± 4.12 | 111.67 ± 2.75 | 115.35 ± 1.37 | 104.42 ± 1.64 | 0.55 ± 0.06 | 0.52 ± 0.05 | 0.61 ± 0.06 | 0.62 ± 0.05 | 106.66 ± 26.09 | 107.42 ± 24.18 | 134.85 ± 24.45 | 184.99 ± 18.96 | - | - | - | - |
| Bagger *et al* (verapamil) | 103.80 ± 11.51 | 104.10± 12.98 | 103.80 ± 11.51 | 102.50 ± 12.49 | 0.59 ± 0.18 | 0.55 ± 0.13 | 0.59 ± 0.18 | 0.57 ± 0.13 | 102.60 ± 58.35 | 100.70 ± 42.41 | 102.60 ± 58.35 | 149.80 ± 104.71 | 40.10 ± 23.34 | 44.90 ± 20.87 | 40.10 ± 23.34 | 57.80 ± 39.61 |
| Robert *et al* (captopril) | 132.80 ± 11.18 | 127.40 ± 12.07 | 132.80 ± 11.18 | 117.40 ± 13.41 | - | - | - | - | 231.46 ± 125.84 | 228.22 ± 148.02 | 231.46 ± 125.84 | 229.22 ± 143.10 | 145.00 ± 88.55 | 145.12 ± 86.35 | 145.00 ± 88.55 | 148.66 ± 70.43 |
| Robert *et al* (atenolol) | 132.80 ± 11.18 | 127.40 ± 12.07 | 132.80 ± 11.18 | 118.80 ± 10.73 | - | - | - | - | 231.46 ± 125.84 | 228.22 ± 148.02 | 231.46 ± 125.84 | 187.64 ± 104.29 | 145.00 ± 88.55 | 145.12 ± 86.35 | 145.00 ± 88.55 | 124.31 ± 84.16 |
| Robert *et al* (labetolol) | 132.80 ± 11.18 | 127.40 ± 12.07 | 132.80± 11.18 | 116.00 ± 12.96 | - | - | - | - | 231.46 ± 125.84 | 228.22 ± 148.02 | 231.46 ± 125.84 | 155.11 ± 94.18 | 145.00 ± 88.55 | 145.12 ± 86.35 | 145.00 ± 88.55 | 109.07 ± 63.55 |
| Robert *et al* (pindolol) | 132.80 ± 11.18 | 127.40 ± 12.07 | 132.80 ± 11.18 | 119.10 ± 12.52 | - | - | - | - | 231.46 ± 125.84 | 228.22 ± 148.02 | 231.46 ± 125.84 | 157.41 ± 89.44 | 145.00 ± 88.55 | 145.12 ± 86.35 | 145.00 ± 88.55 | 100.10 ± 68.42 |

**Supplementary Table 1**

**Table 1: Blood pressure and measure of PAD severity reported in studies included in this meta-analysis.**

Results are expressed as mean ± SD. The mathematical approach taken for obtaining these data is given in S2 table. Mean arterial pressure was defined from the following formula: MAP = [(2*DBP) + SBP] / 3. Different anti-hypertensives were used in the trials: Overlack trial – perindopril, Shahin et al – ramipril, Zankl et al – telmisartan, Bagger et al – verapamil, Robert et al – captopril, atenolol, labetolol and pindolol. Abbreviations: DBP- diastolic blood pressure, MAP – mean arterial pressure, PI – post-intervention, and SBP – systolic blood pressure.
