# Supplementary material for: Effect of blood pressure lowering medications on leg ischemia in peripheral artery disease patients: A meta-analysis of randomised controlled trials
Source: PLoS One. 2017 Jun 2;12(6):e0178713. doi: 10.1371/journal.pone.0178713 (PMC5456103; doi:10.1371/journal.pone.0178713)
Supplement: S2 Table — (DOCX) [file pone.0178713.s006.docx]

**Supplementary Table 2**

**Table 1: Calculations involved in the included trials**

1. **Overlack *et al.***

| The following formulas were used to calculate MAP:  MAP = (2 * diastolic blood pressure) + systolic blood pressure / 3 🡪 ① [1]  Standard deviation (SD) = SEM (standard error of mean) * √n, where n is the sample size of the group 🡪 ② [2]  Variance = SD^2^ 🡪 ③ [2]  Sum of squares (SS) = variance * (n-1) 🡪 ④ [2]  Total sum of squares (TSS) = [(2 * SS of diastolic blood pressure) + SS of systolic blood pressure / 3] ⑤ [2]  Residual variance (RV) =TSS/ (n-1) 🡪 ⑥ [2]  Pooled SD = √ RV 🡪 ⑦ [2] | |
| --- | --- |
| **Perindopril (baseline)** | **Placebo (baseline)** |
| SBP = 160.0 ± 4.4  DBP = 99.5 ± 0.9  MAP = 119.66 (using equation ①) | SBP = 157.2 ± 2.8  DBP = 98.9 ± 0.8  MAP = 118.33 (using ①) |
| Calculation for SEM for MAP:  SEM of SBP = 4.4,  SD = 22.4 (n=26), (using ②)  variance = 501.8, (using ③)  sum of squares (SS) =12545, (using ④)  SEM of DBP =0.9,  SD = 4.6,  Variance = 21.2  SS= 530  Total SS = 4535, (using ⑤)  Residual variance = (TSS/(n-1)) = 181.4, (using ⑥)  Pooled SD = 13.5, (using ⑦) | Calculation for SEM for MAP:  SEM of SBP = 2.8,  SD = 14.812 (n=28),  variance = 219.39,  sum of squares (SS) =5923.53  SEM of DBP =0.8,  SD = 4.232,  Variance = 17.9  SS= 483.3  Total SS = 2296.71  Residual variance = (TSS/(n-1)) = 85.06  Pooled SD = 9.22 |
| **Perindopril (post-intervention)** | **Placebo (post-intervention)** |
| SBP = 147.7 ± 4.4  DBP = 89.7 ± 1.5  MAP = 109.03 | SBP = 154.3 ± 3.9  DBP = 95.0 ± 1.2  MAP = 114.76 |
| Calculation for SEM for MAP:  SEM of SBP = 4.4,  SD = 22.4 (n=26),  variance = 501.8,  sum of squares (SS) =12545  SEM of DBP =1.5,  SD = 7.64,  Variance = 58.4  SS= 1460  Total SS = 5155  Residual variance = (TSS/(n-1)) = 206.2  Pooled SD = 14.35 | Calculation for SEM for MAP:  SEM of SBP = 3.9,  SD = 20.63 (n=28),  variance = 425.59,  sum of squares (SS) =11490.9  SEM of DBP =1.2,  SD = 6.348,  Variance = 40.29  SS= 1087.83  Total SS = 4555.53  Residual variance = (TSS/(n-1)) = 168.72  Pooled SD = 12.98 |
| **Perindopril**  **MAP (baseline) = 119.66 ± 13.50**  **MAP (post intervention) = 109.03 ± 14.35** | **Placebo**  **MAP (baseline) = 118.33 ± 9.22**  **MAP (post intervention) = 114.76 ± 12.98** |
| SBP – systolic blood pressure, DBP – diastolic blood pressure, SBP and DBP is expressed as mean (SEM), MAP – mean arterial pressure. | |

1. **Shahin *et al.***

| Following formulas were used in the below calculations:  Mean = (q1 + median + q3)/ 3, from median (inter-quartile range, IQR) 🡪 ⑧ [3]  SD = (q3 – q1)/ ƞ(n), where n = sample size, (SD from IQR) 🡪 ➈ [3] | |
| --- | --- |
| **Ramipril (baseline)** | **Placebo (baseline)** |
| MAP = 99 ± 3 (mean ± SEM), n = 12  **MAP = 99 ± 10.39 (mean ± SD)** (using ②) | MAP = 105 ± 3 (mean ± SEM), n = 17  **MAP = 105 ± 12.37 (mean ± SD)** |
| **ABPI = 0.59 ± 0.19 (mean ± SD)** | **ABPI = 0.66 ± 0.16** |
| MWD = 137 (110 – 213) (median, IQR)  Mean = 153.33 (using ⑧)  SD = 85.40, where ƞ(n) = 1.206 (using ➈)    **MWD = 153.33 ± 85.40** | MWD = 143 (72-213)  Mean = 142.66  SD = 113.80, where ƞ(n) = 1.239    **MWD = 142.66 ± 113.80** |
| PFWD = 81 (48 – 114) (median, IQR)  Mean = 81  SD = 54.73  **PFWD = 81 ± 54.73** | PFWD = 94 (32 – 163) (median, IQR)  Mean = 96.33  SD = 105.73  **PFWD = 96.33 ± 105.73** |

| **Ramipril (post intervention), 2 weeks** | **Placebo (post intervention), 2 weeks** |
| --- | --- |
| MAP = -7.0 ± 2 (mean change ± SEM), n = 12  MAP (post intervention) = 92 (mean)  Assuming that there is no correlation between the change of MAP and baseline MAP, converting the SD's to variances, adding the variances and then taking the square root of the sum will give us the post intervention SD.  SD at baseline = 10.39  Variance = 107.95 (using ③)  SEM of change = 2  SD of change = 6.93 (using ②)  Variance of change = 48.025 (using ③)  Sum of variances = 155.98  Pooled SD = 12.48 (using ⑦)  **MAP = 92 ± 12.48** | MAP = -0.5 ± 2 (mean change ± SEM), n = 17  MAP (post intervention) = 104.5 (mean)  Assuming that there is no correlation between the change of MAP and baseline MAP, converting the SD's to variances, adding the variances and then taking the square root of the sum will give us the post intervention SD.  SD at baseline = 12.37  Variance = 153.02  SEM of change = 2  SD of change = 8.246  Variance of change = 68  Sum of variances = 221.02  Pooled SD = 14.86  **MAP = 104.5 ± 14.86** |
| MWD = + 34.49 ± 18.33 (mean change and SEM)  MWD (total) = 153.33 + 34.49 = 187.82  Using the above formulas the final SD can be computed:  SD at baseline = 85.40  Variance = 7293.16  SEM of change = 18.33  SD of change = 63.5  Variance of change = 4032.25  Sum of variances = 11325.41  Pooled SD = 106.42  **MWD = 187.82 ± 106.42** | MWD = +10.20 ± 17.30 (mean change and SEM)  MWD (total) = 152.86  Using the above formulas the final SD can be computed:  SD at baseline = 113.80  Variance = 12950.44  SEM of change = 17.30  SD of change = 71.32  Variance of change = 5086.54  Sum of variances = 18036.98  Pooled SD = 135.30  **MWD = 152.86 ± 134.30** |

| **Ramipril (post intervention), 24 weeks** | **Placebo (post intervention), 24 weeks** |
| --- | --- |
| MAP = -9.0 ± 2 (mean change ± SEM), n = 12  MAP (post intervention) = 90 (mean)  As above,  SD at baseline = 10.39  Variance = 107.95  SEM of change = 2  SD of change = 6.93  Variance of change = 48.025  Sum of variances = 155.98  Pooled SD = 12.48  **MAP = 90 ± 12.48** | MAP = +2 ± 2 (mean change ± SEM), n = 17  MAP (post intervention) = 107 (mean)  As above,  SD at baseline = 12.37  Variance = 153.02  SEM of change = 2  SD of change = 8.246  Variance of change = 68  Sum of variances = 221.02  Pooled SD = 14.86  **MAP = 107 ± 14.86** |
| ABPI = +0.03 ± 0.08 (mean change and SD)  ABPI (total) = 0.62  As above,  SD at baseline = 0.19  Variance = 0.0361  SD of change = 0.08  Variance= 0.0064  Sum of variances = 0.0425  Pooled SD = 0.20  **ABPI = 0.62 ± 0.20** | ABPI = + 0.02 ± 0.18 (mean change and SD)  ABPI (total) = 0.68  As above,  SD at baseline = 0.16  Variance = 0.0256  SD of change = 0.18  Variance= 0.0324  Sum of variances = 0.058  Pooled SD = 0.24  **ABPI = 0.68 ± 0.24** |
| MWD = + 153.10 ± 23.47 (mean change and SEM)  MWD (total) = 153.33 + 153 = 187.82  Using the above formulas the final SD can be computed:  SD at baseline = 85.40  Variance = 7293.16  SEM of change = 23.47  SD of change = 81.31  Variance of change = 6611.32  Sum of variances = 13904.48  Pooled SD = 117.92  **MWD = 306.43 ± 117.92** | MWD = +22.91 ± 19.1 (mean change and SEM)  MWD (total) = 165.57  Using the above formulas the final SD can be computed:  SD at baseline = 113.80  Variance = 12950.44  SEM of change = 19.1  SD of change = 78.74  Variance of change = 6199.99  Sum of variances = 19140.43  Pooled SD = 138.38  **MWD = 165.57 ± 138.38** |
| PFWD = + 144.09 ± 23.73 (mean change, SEM)  PFWD (total) = 225.09  Using the above formulas the final SD can be computed:  SD at baseline = 54.73  Variance = 2995.37  SEM of change = 23.73  SD of change = 82.20  Variance of change = 6756.84  Sum of variances = 9752.21  Pooled SD = 98.75  **PFWD = 225.09 ± 98.75** | PFWD = + 22.53 ± 21.9  PFWD (total) = 116.53  Using the above formulas the final SD can be computed:  SD at baseline = 105.73  Variance = 11178.83  SEM of change = 21.9  SD of change = 90.29  Variance of change = 8152.28  Sum of variances = 19331.11  Pooled SD = 139.03  **PFWD = 116.53 ± 139.03** |
| ABPI, MWD and PFWD post intervention values were extrapolated from graphs and used for calculations. Abbreviations: ABPI – ankle brachial pressure index, MAP – mean arterial pressure, MWD – maximum walking distance, PFWD – pain free walking distance, SEM – standard error of mean, q1 q3 – interquartile range (IQR), SD (standard deviation) = SEM * √n, variance = SD^2^. | |

**Reference**

1. Cywinski J, Tardieu B. The essentials in pressure monitoring: Blood and other body fluids: Martinus Nijhoff Medical Division; 1980.
2. Higgins J. Green S. Cochrane handbook for systematic reviews of interventions version 5.1. 0. The Cochrane Collaboration, 2011. 2013.
3. Wan X, Wang W, Liu J, Tong T. Estimating the sample mean and standard deviation from the sample size, median, range and/or interquartile range. BMC medical research methodology. 2014;14(1):135.
